# COVID-19 Prevalence in Nations with Normal Body Mass Index (BMI): Implications of Artificial Intelligence (AI) in Healthcare

**Published:** 2025-04-09

**Authors:** Clement G. Yedjou, Richard Long, Victor Eno, Jinwei Liu, Shannon Bright Smith, Pierre Ngnepieba, Kwasi Densu, Monique MsCallister, Lekan Latinwo, Paul B. Tchounwou

**Affiliations:** 1 Department of Biological Sciences, College of Science and Technology, Florida Agricultural and Mechanical University, Florida, United States of America; 2 Department of History and Political Science, College of Social Sciences, Arts, and Humanities (CSSAH), Florida Agricultural and Mechanical University, Florida, United States of America; 3 Department of Computer and information Sciences, College of Science and Technology, Florida Agricultural and Mechanical University, Florida, United States of America; 4 School of Nursing, Florida Agricultural and Mechanical University, Florida, United States of America; 5 Department of Biological Sciences, Tennessee State University, Nashville, United States of America; 6 RCMI Center for Urban Health Disparities Research and Innovation, Morgan State University, Baltimore, United States of America

**Keywords:** COVID-19, Dietary patterns, Obesity, Artificial intelligence, Machine learning, Public health, Health care systems

## Abstract

**Introduction:**

Coronavirus disease 2019 (COVID-19) has had a profound impact globally, causing the death of millions of people and deeply affecting socio-psychological, human health, and economic systems, with some nations bearing a disproportionate burden. Despite obesity having been established as one of the major risk factors of COVID-19 severity and other degenerative diseases, the effects that dietary pattern intake plays in COVID-19 outcomes remain poorly understood. The goal of this study is to look into the connection between eating habits, the number of non-obese and obese people, and COVID-19 outcomes in countries with populations exhibiting normal Body Mass Index (BMI), which is an indicator of obesity.

**Methods:**

The analysis includes data from 170 countries. From the 170 countries, we focused on 53 nations where the average, BMI falls within the normal range (18.5 to 24.9). A subset of 20 nations was selected for a more detailed examination, comprising 10 nations with the lowest BMI values within the normal range (18.5–19.8) and 10 nations with the highest BMI values within the normal range (23.5–24.9). We used Artificial Intelligence (AI) and Machine Learning (ML) applications to evaluate key metrics, including dietary patterns (sugar and vegetable intake), obesity prevalence, incidence rate, mortality rate, and Case Fatality Rate (CFR).

**Results:**

The results demonstrate a significant correlation between higher obesity prevalence and increased COVID-19 severity, evidenced by elevated incidence, mortality, and CFRs in countries like North Macedonia and Italy. In contrast, nations such as Iceland and New Zealand with well-established healthcare systems revealed low mortality rate and case fatality rate despite variations in dietary habits. The study also revealed that vegetable consumption appears to provide a slight to significant protective effects, suggesting that dietary patters alone do not consistently predict COVID-19 Outcomes.

**Conclusion:**

Data generated from this study showed the crucial role of healthcare infrastructure along with the testing capacity and data reporting in influencing the success of pandemic responses. It also highlights the need of integrating public health strategies, which focus on obesity management and improvement of healthcare preparedness. In addition, AI-driven predictive modeling offers valuable insights that may guide pandemic response efforts in the future, thereby enhancing global health crisis management and mitigating the impact of future health emergencies.

## INTRODUCTION

Coronavirus disease 2019 (COVID-19) has had a profound impact globally, causing the death of millions of people and deeply affecting socio-psychological, human health, and economic systems, with some nations bearing a disproportionate burden [1–3]. For example, in the United States of America (USA), COVID-19 mortality has disproportionately caused the death of Black Americans who experienced severe clinical outcomes compared to other racial groups [4,5]. The previous meta-analysis, which comprised of fifty (50) scientific studies, demonstrated that Black American patients have a higher risk of seeking Intensive Care Unit (ICU) admission due to COVID-19 compared to White patients [6,7]. In addition, a meta-analysis that reviewed forty-five (45) published research articles observed that race is linked with worse COVID-19 outcomes, likely driven by a higher prevalence of comorbidities among racial and ethnic underserved populations [8]. The racial disparities highlighted here showed the major role of social determinants of health, including genetic, lifestyle and environmental factors, socioeconomic status, and healthcare access in influencing disease severity and adverse health outcomes.

Obesity is a chronic health condition that is linked to several comorbidities such as metabolic syndrome, diabetes, and cardiovascular diseases. This disease is not only a major public health challenge but also a significant predictor of COVID-19 severity [9]. Currently, obesity is recognized as a disease of pandemic due to its widespread nature throughout the world [10]. Studies demonstrated that people with obesity are more susceptible to COVID-19 infection and have a higher risk for severe outcomes, including mortality, compared to the general population [11,12]. Other scientific studies have highlighted that people with obesity face increased risks of hospitalization, ICU admission, mechanical ventilation, and death from COVID-19 among people with obesity [13,14]. The inflammatory and immune disjunction associated with excess adiposity further exacerbates these risks. Obesity’s prevalence is alarmingly high, ranging from 3.7% in Japan to 38.2 in the United States [15,16]. Furthermore, factors such as limited access to primary care, poor lifestyle choices, lack of information, insufficient health insurance, geographic location, and lower education levels contribute to disparities in COVID-19 outcomes within affected populations [17,18]. Therefore, understanding the impact of obesity on COVID-19 mortality is crucial.

Emerging research data demonstrates the vital role of a healthy diet in boosting the human body’s immune system and lowering the risks of diseases. Several studies indicated that nutrients rich in fruits, vegetables, whole grains, and lean proteins enhance the immune responses and lower inflammation levels, potentially aiding in the prevention and/or management of COVID-19 severe [19–22]. Recent research in our laboratory has demonstrated that edible medicinal plants and vitamins-such as *Allium sativum* (garlic), ascorbic acid (vitamin C), curcumin, *Nigella sativa* (black seed), vitamin D, and *Zingiber officinale* (ginger)-possess novel therapeutic effects for preventing and treating COVID-19 [23]. These substances share eight common pharmacological properties, including antibacterial, anticancer, anti-inflammatory, immunomodulatory, antioxidant, antifungal, antimutagenic, and antiviral activities [23]. In addition, other studies have shown that a healthy diet rich in fruits and vegetables enhance the immune system and may prevent diseases such as cardiovascular diseases, diabetes, and cancer [24]. However, while the role of obesity as a risk factor for severe COVID-19 outcomes is well established, the impact of dietary patterns on COVID-19 severity remains less clear.

The objective of this present research study is to explore the connection between dietary habits, obesity prevalence, and COVID-19 outcomes in populations with normal BMI levels. We utilized Artificial Intelligence (AI) and Machine Learning (ML) applications to evaluate key metrics, including obesity prevalence, dietary patterns (sugar and vegetable intake), incidence rates, mortality rates, Case Fatality Rates (CFR), and uncovered actionable insights that can inform public health interventions. Previous studies have showed that AI and ML have the potential to revolutionizing healthcare.

These innovative tools are able to identify patterns that guide disease prevention strategies. In addition, AI-driven predictive modeling offers valuable insights that may guide pandemic response efforts in the future, thereby enhancing global health crisis management and mitigating the impact of future health emergencies.

## MATERIALS AND METHODS

### Study design and data sources

The analysis includes data from 170 countries. From the 170 countries, we focused on 53 nations where the average BMI falls within the normal range (18.5 to 24.9). A subset of 20 nations was selected for a more detailed examination, comprising 10 nations with the lowest BMI values within the normal range (18.5–19.8) and 10 nations with the highest BMI values within the normal range (23.5–24.9). We used Artificial Intelligence (AI) and Machine Learning (ML) applications to evaluate key metrics, including dietary patterns (sugar and vegetable intake), obesity prevalence, incidence rate, mortality rate, and Case Fatality Rate (CFR).

The data used for our study were sourced from various trusted organizations, including:

- John Hopkins Center for Systems Science and Engineering (CSSE): Here, we collected data for COVID-19 confirmed, deaths, recovered, and active cases.
- Food and Agricultural Organization of the United Nations (FAO). Here, we collected data for different food supply quantities, nutrition values, and obesity.
- USDA Center for Nutrition Policy and Promotion. Diet intake information
- Population Reference Bureau (PRB). Here, we collected data for population from each nation.

### Variables for analysis

The different variables selected for the present study include:

- Dietary patterns including average sugar and sweeteners intake express in kilogram (kg) in each country and average vegetable intake expresses in kilogram (kg) in each nation.
- Body Mass Index (BMI): the average BMI, which is used as an indicator of obesity prevalence in the population.
- Incidence expresses in percentage of the population that has been diagnosed with COVID-19 in each nation.
- Mortality expresses in percentage of the people who died due to COVID-19 in relation to the total number of reported cases in each nation.
- Case Fatality Rate (CFR): The proportion of individuals diagnosed with COVID-19 who die from the disease, typically expressed as a percentage.

Selection and exclusion criteria

- Selection criteria: We selected nations and categorized them based on their national average body mass index (BMI) to on the population group with normal BMI range.
- Exclusion criteria: We eliminated nations with missing, incomplete, or unreliable COVID-19 reported data. In addition, we excluded the population group that the BMI fell outside the normal range which is either lower or higher.

### Data processing and AI/ML approach

For this experimental procedure, we used Artificial Intelligence (AI) and Machine Learning (ML) applications to evaluate key metrics, including dietary patterns (sugar and vegetable intake), obesity prevalence, incidence rate, mortality rate, and Case Fatality Rate (CFR). Recent studies have showed an emerging integration of AI in healthcare due to its potential benefits, technological advancements, and availability of extensive datasets [25,26].

AI tools, such as those used in diagnostics, such as X-rays and MRIs, have shown remarkable accuracy in interpreting medical images, often surpassing traditional methods [27–29]. Furthermore, machine learning, a subset of AI, plays a key role in personalizing treatment by tailoring approaches to individual patient profiles, thus optimizing therapeutic results [30]. In addition, Excel was employed to replicate and validate the data generated by the AI/ML analysis.

### Ethical considerations

To maintain ethical considerations, we used publicly available dataset to ensure compliance with data protection regulations in our study. In addition, to maintain privacy and ethical research standards, we did not analyze individual-level patient data.

### Statistical analysis

The study utilized four (4) approaches for the statistical analysis.

- Comparative analysis: Nations with similar BMIs but varying pandemic outcomes were compared to assess the impact of healthcare infrastructure and dietary habits.
- Descriptive statistics: We performed initial Exploratory Data Analysis (EDA) to summarize the major characteristics of the dataset, including the mean value, standard deviation, median, and frequency distributions.
- Correlation analysis: To identify and measure correlations between different variables (features) including dietary patterns, obesity, and COVID-19, we used we used Pearson’s and Spearman’s Correlation tests.
- Multivariate progression models: We used this method for confounding variables, including healthcare systems capacity and socioeconomic factors.

All data analyses were conducted using programming languages including python and RStudio. Excel analysis was also used. P-value<0.05 were considered significant.

## RESULTS

Our results are summarized in Table 1 below based on the analysis of the same variables for nations with populations exhibiting normal BMI. It highlights data on dietary patterns (including sugar and sweeteners, vegetables), obesity prevalence, and COVID-19 metrics (such as incidence rate, mortality rate, and case fatality rate) for different nations.

### Dietary patterns and COVID-19 metrics

Our result showed that high sugar intake, as observed in nations such as Honduras (5.96 kg) and Nicaragua (5.70 kg), did not consistently correlate with worse COVID-19 outcomes. For example, Honduras experienced a moderate incidence rate of 1.56% and a low mortality rate of 0.038%, whereas Israel, which had relatively low sugar intake (1.77 kg), reported a higher incidence rate at 7.44%. In contrast, the consumption of high vegetable in nations such as Nepal (9.28 kg) and Niger (10.61 kg) show a lower incidence rate of 0.91% and 0.02%, respectively, highlighting a greater resilience to severe outbreaks. However, Ireland, with a moderate levels of vegetable consumption (4.95 kg) has a higher incidence rate of 4.05%, suggesting that other factors may play a significant role in shaping COVID-19 outcomes.

### Obesity and COVID-19 metrics

Our results demonstrate that obesity prevalence positively correlate with poorer health outcomes. For example, North Macedonia, with a normal Body Mass Index (BMI), an indicator of obesity with a value of 24.5, experienced high incidence rate (4.55%) and CFR (3.08%). In contrast, Japan, where the normal BMI is 19.8, reported lower rates (incidence: 0.32%, CFR: 1.58%). Niger, with a normal BMI of 24.4, had a notably low incidence rate (0.02%) and mortality (0.001%), which can likely be associated with limited testing or healthcare access rather than reflecting the true prevalence of obesity in the population.

**Figure 1:**
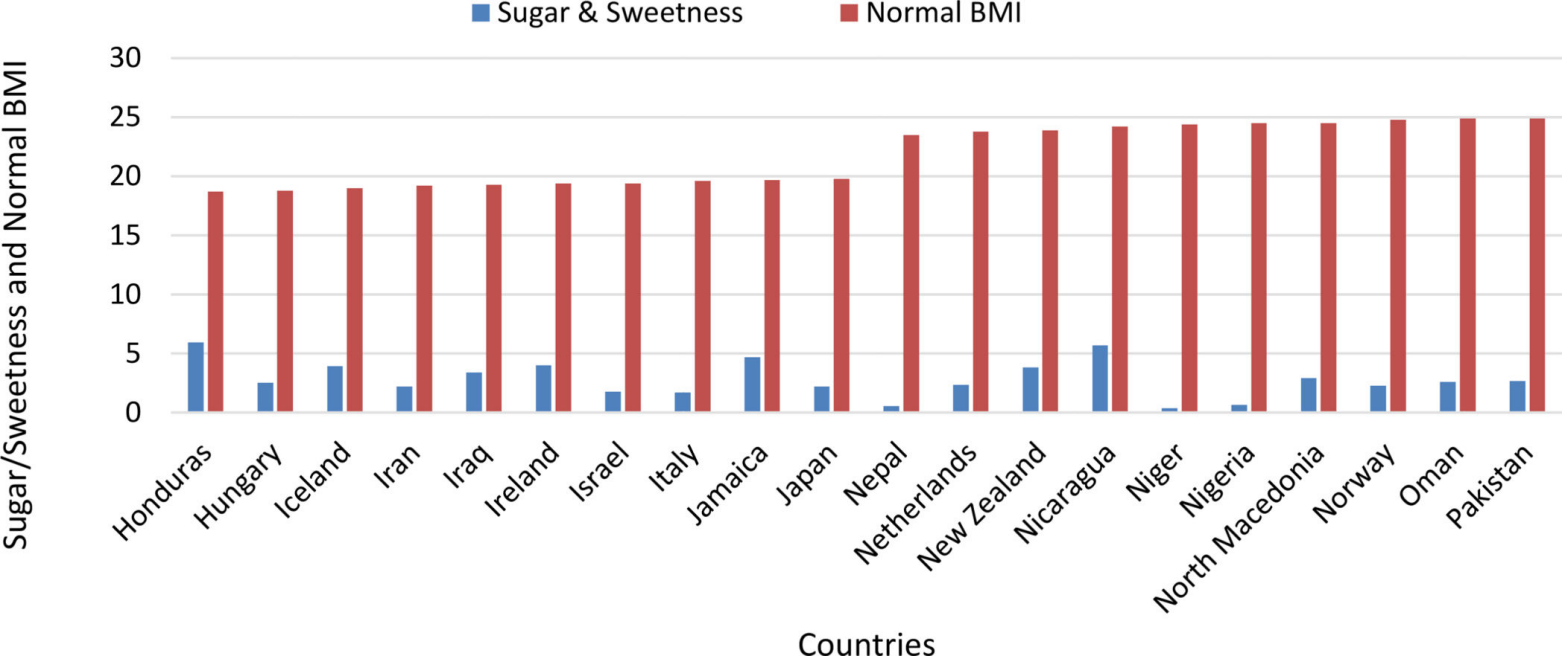
Comparison of sugar consumption and normal BMI.

When evaluating the intake of sugar, a known risk factor for obesity, among people with a normal BMI, our data revealed complex findings. Both low and high sugar intake were linked to distinct impacts on health outcomes, as illustrated in Figure 1.

**Figure 2:**
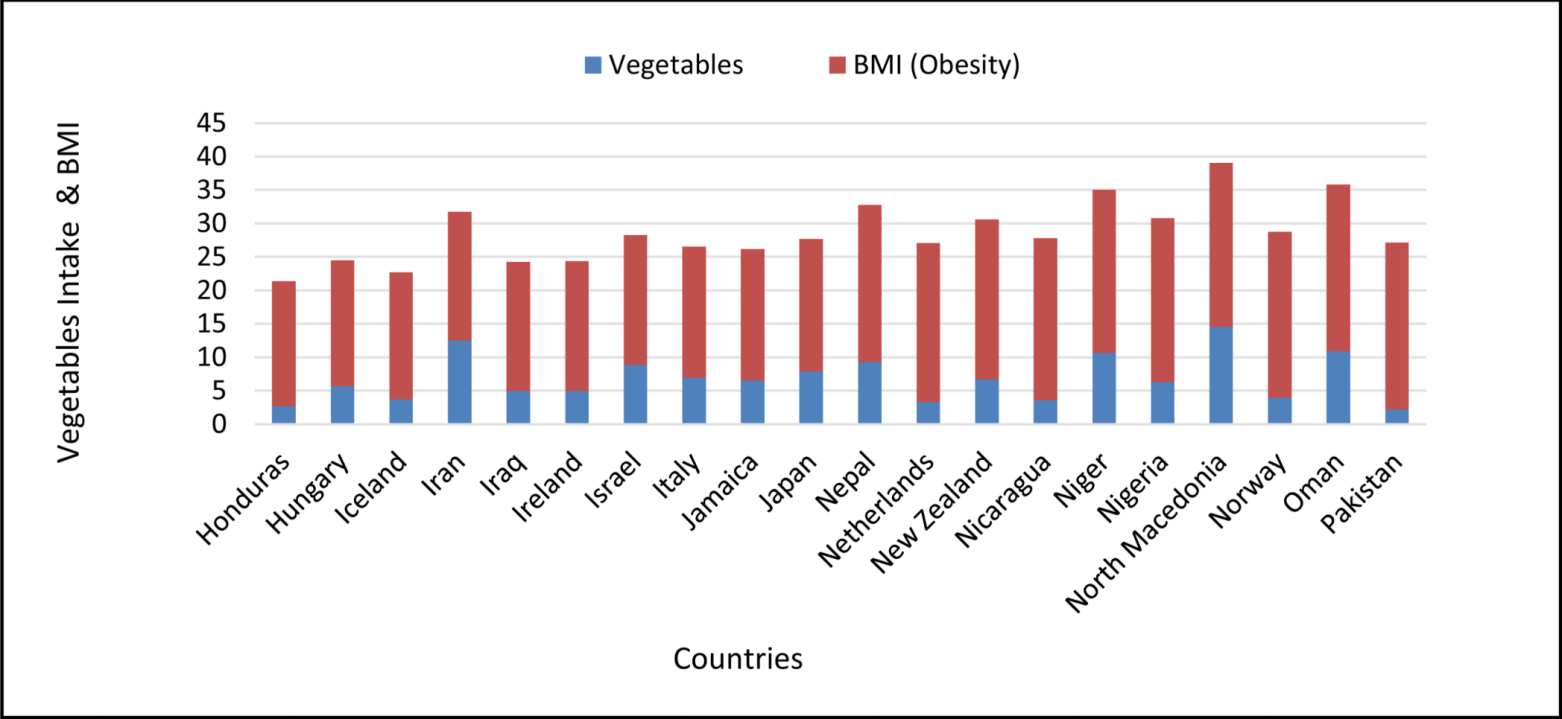
Comparison of vegetables consumption and normal BMI.

When comparing vegetable intake with normal BMI, our analysis shows that higher vegetables consumption is associated with a slight to significant reduction in BMI, which is a key indicator of obesity (Figure 2).

### Incidence rate

With regard to incidence rate, our results demonstrate that nations with the highest incidence rates, such as Israel (7.44%), the Netherlands (5.82%), and Italy (4.35%), typically benefit from well-advanced healthcare systems and robust testing centers.

These finding highlights that the elevated incidence rates may be more indicative of early detection capabilities rather than a higher rate of disease transmission. Conversely, nations with exceptionally low incidence rates, such as Niger (0.02%) and Nicaragua (0.10%), may be experiencing underreporting, potentially due to limited testing centers and surveillance resources.

### Mortality rate

In general, our results reveal that mortality rates remain relatively low across most nations, with the highest observed rates in Italy (0.151%) and North Macedonia (0.140%). The high incidence rates observed for Italy and Macedonia (4.35% and 4.55%, respectively), can be linked to the higher mortality rates.

In comparing to other countries, nations such as New Zealand (0.001%) and Niger (0.001%), show low mortality rates, suggesting effective pandemic management strategies and demographics characterized by the youngest populations who are healthy or have low levels of underlying health conditions.

### Case Fatality Rate (CFR)

Our results show that case fatality rate significant variability, with the highest rates observed in Niger (3.59%), Iran (4.00%), and North Macedonia (3.08%). These high elevated CFRs may be linked to healthcare infrastructure deficiencies or delays in COVID-19 treatment.

In contrast, nations including Iceland (0.48%) and Israel (0.74%) report considerably lower CFRs, which may be attributed to advanced healthcare systems and timely medical interventions.

### Key observations and findings

**Figure 3:**
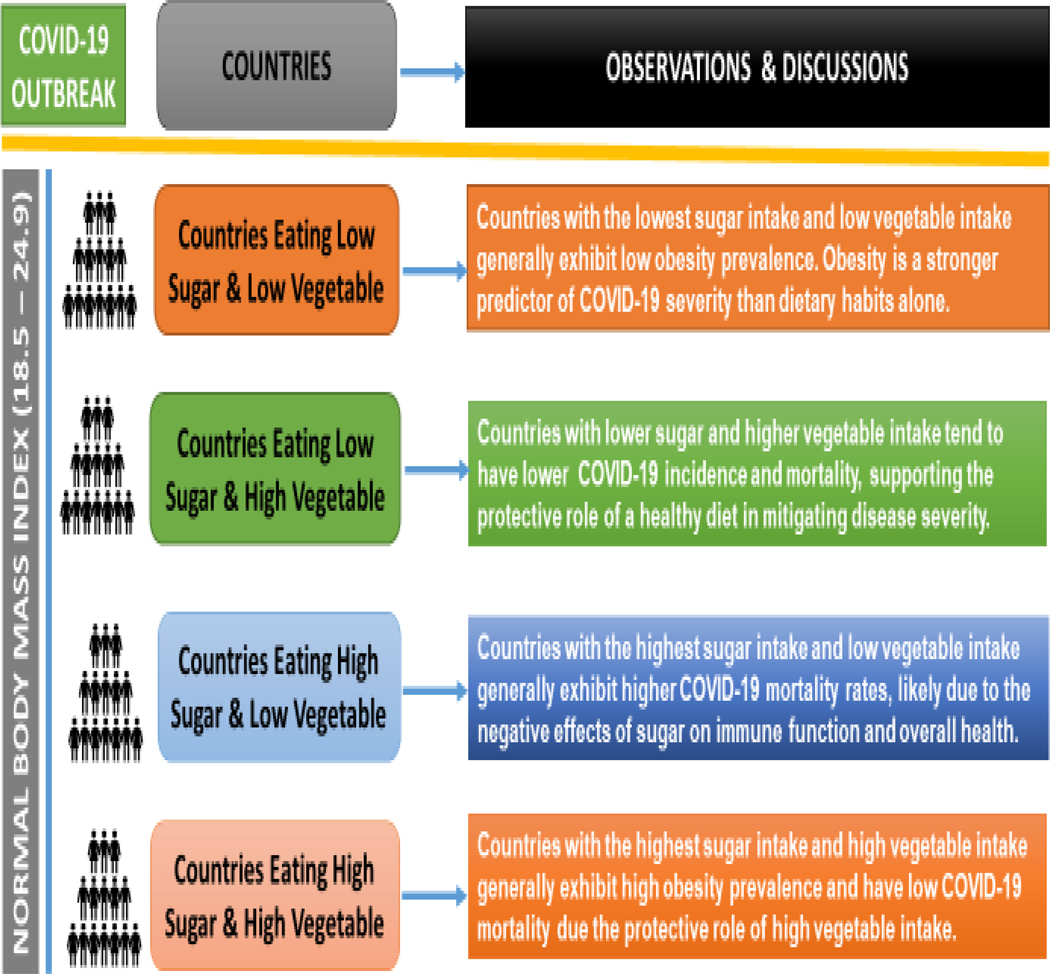
Influence of sugar and vegetables intake on COVID-19 outcomes.

After our comprehensive analysis, we observed several key insights, summarized in Figure 3. As seen in Figure 3, it highlights the distinct impacts of dietary patterns (sugar and vegetable intake) on COVID-19 outcomes, illustrating the notable influence of these dietary factors on the progression and severity of the disease.

### Healthcare system and its impact

We found that nations with robust healthcare infrastructures, including Iceland and New Zealand, have reported exceptionally low rate of COVID-19 mortality (0.008% and 0.001%, respectively) and case fatality rate (0.48% and 1.08%, respectively). These outcomes persist despite differing dietary patterns, highlighting the critical role of healthcare systems in mitigating the severity of COVID-19.

## DISCUSSION

Scientific reports demonstrated that obese people are more vunerable to COVID-19 infection and have a higher risk for severe outcomes, including mortality, compared to non-obese people [11,12]. Several published articles have showed that obesity is associated with cardiovascular diseases, diabetes, metabolic syndrome, and increase risk of developing hypertension. These underlying diseases are linked to an increase risk of severe COVID-19 onset [31–33]. COVID-19 onset has caused a major threat to the medical community and healthcare systems worldwide.

Excessive sugar consumption has been linked to poorer diet quality, increased caloric intake, and a higher prevalence of obesity [34]. In contrast, we found in our study that high intake of sugar observed in nations like Honduras (5.96 kg), and Nicaragua (5.70 kg) did not correlate with worse COVID-19 outcomes. For instance, Honduras with a high sugar intake, had a relatively low mortality rate of 0.038%, despite a moderate incidence rate of 1.56%. On the other end, Israel with a lower sugar intake (1.77 kg), experienced the highest incidence rate of 7.44%. Although obesity is linked to worse COVID-19 outcomes, our data reveal more complex relationships.

As example, country like North Macedonia, with a normal Body Mass Index (BMI) of 24.5, has a higher incidence (4.55%) and Case Fatality Rate (CFR) (3.08%), whereas Japan, with a BMI of 19.8, exhibits a lower rate (incidence: 0.32%, CFR: 1.58%). Interestingly, Niger, with a BMI of 24.4, reported a lower incidence rate (0.02%) and mortality rate (0.001%), probably due to limited testing or lack of healthcare access rather than an accurate reflection of disease prevalence. Obesity is a critical e risk factor for COVID-19 and other metabolic diseases that is easily modifiable naturally. Addressing obesity through lifestyle interventions and other measures could help mitigate transmission and reduce the impact of the SARS-CoV-2 pandemic [35]. Weight loss, achieved through dietary adjustments, physical activity, and both surgical and non-surgical interventions, is essential for reducing the adverse effects of obesity and managing its associated comorbidities [36–38].

Our study also revealed that high vegetable intake (e.g., Nepal: 9.2 kg; Niger: 10.61 kg) was correlated with low COVID-19 incidence rates (0.91% and 0.02%, respectively), suggesting potential resilience against severe outbreaks. However, countries like Ireland, with a vegetable consumption of 4.95 kg with a high incidence rate (4.05%), indicate that other factors also play significant roles. A substantial body of research supports the idea that healthy eating patterns, combined with regular physical activity, are essential for promoting overall health and reducing the risk of diseases such as cancer and diabetes. Nonetheless, many countries, especially wealthier nations, fall short for meeting the recommended levels of vegetable and fruit intake. For instance, most adults in the United States do not adhere to the recommended dietary guidelines for vegetables and fruits [39–41]. In a recent research conducted in our laboratory, we demonstrated that edible medicinal plants (such as black seed, curcumin, garlic, and ginger) and vitamins (including vitamin C and D) exhibit antiviral activities, with their individual intake showing promise for the prevention and/or control of COVID-19 [23]. Beyond their antiviral properties these edible medicinal plants and vitamins share eight pharmacological effects: Anti-bacterial, anti-cancer, anti-fungal, anti-inflammatory, anti-mutagenic, antioxidant, anti-viral, and immunomodulatory [23]. We conclude that, these selected edible medicinal plants and vitamins possess anti-viral properties that are more likely to disrupt the SARS-CoV-2 replication cycle, enhance the human immune system, and promote overall health [23].

Regular exercise and balanced diet play a crucial role in maintaining a normal healthy lifestyle. By adopting these practices, people not only enhance their personal health, but also contribute to the wellbeing of their communities, ensuring that everyone is equipped to safeguard their health and contribute to collective protection. In addition, actions including behavioral modification, engaging in physical activity, and eating a nutritious diet can significantly lower virus transmission, curtail its propagation, and enhance outcomes for both individuals and communities. The World Health Organization (WHO) has identified physical inactivity as one the major risk factor contributing to global mortality. Their guidelines emphasize that even moderate physical activity offers benefits over inactivity, and higher levels of physical activity yields even more favorable health outcomes [42–44].

Additionally, our findings demonstrate that countries with advanced healthcare systems, such as Iceland and New Zealand, experienced low mortality rates (0.008% and 0.001%, respectively) and CFRs (0.48% and 1.08%, respectively) despite differing dietary habits. This highlights the critical role of healthcare infrastructure in reducing the severity of COVID-19. Nations with advanced healthcare systems offer significant advantages, including timely diagnoses, early detection of disease, and personalized treatment plans. They also expand access to care through telehealth, enhance patient safety by minimizing medical errors, and improve cost-efficiency by optimizing resource allocation. Furthermore, these robust healthcare systems support the effective management of chronic conditions, contributing to long-term health outcomes.

## CONCLUSION

Obesity is a chronic health condition that is linked to several comorbidities such as metabolic syndrome, diabetes, and cardiovascular diseases. This disease is not only a major public health challenge but also a significant predictor of COVID-19 severity. Therefore, the present research study underscores the pivotal role of obesity and healthcare infrastructure in transforming COVID-19 outcomes, with dietary patterns playing a secondary role. Findings from our analysis showed that nations with higher rates of normal BMI such as North Macedonia and Italy, are more likely to experience worse COVID-19 outcomes. Nations with advanced healthcare infrastructure such as New Zealand and Iceland, consistently reveal lower mortality rates and case fatality rates, underscoring the critical need for preparedness and access to care. Our research findings also suggest that obesity is a stronger predictor of COVID-19 than dietary habits alone, highlighting the need of for public health initiatives focused weight management. Collectively, our data generated from this study showed the crucial role of healthcare infrastructure along with the testing capacity and data reporting in influencing the success of pandemic responses.

It also highlights the need for integrating public health strategies, which focus on obesity management and improvement of healthcare preparedness. In addition, AI-driven predictive modeling offers valuable insights that may guide pandemic response efforts in the future, thereby enhancing global health crisis management and mitigating the impact of future health emergencies.

## Figures and Tables

**Table 1:** Comparative analysis of dietary patterns and COVID-19 metrics.

Country	Sugar and Sweeteners (kg)	Vegetables (kg)	Normal BMI: (Obesity)	Incidence Rate (%)	Mortality Rate (%)	Case Fatality Rate
Honduras	5.9591	2.6599	18.7	1.56	0.038	2.41
Hungary	2.5433	5.6788	18.8	3.84	0.133	3.47
Iceland	3.9472	3.6874	19	1.64	0.008	0.48
Iran	2.198	12.5207	19.2	1.73	0.069	4.00
Iraq	3.3755	4.9563	19.3	1.58	0.033	2.09
Ireland	4.0147	4.951	19.4	4.05	0.073	1.81
Israel	1.7747	8.8586	19.4	7.44	0.055	0.74
Italy	1.7175	6.9463	19.6	4.35	0.151	3.47
Jamaica	4.6889	6.4772	19.7	0.60	0.013	2.12
Japan	2.2055	7.8669	19.8	0.32	0.005	1.58
Nepal	0.547	9.277	23.5	0.91	0.007	0.75
Netherlands	2.3446	3.2587	23.8	5.82	0.083	1.42
New Zealand	3.8188	6.6889	23.9	0.05	0.001	1.08
Nicaragua	5.7021	3.5808	24.2	0.10	0.003	2.70
Niger	0.3666	10.6117	24.4	0.02	0.001	3.59
Nigeria	0.6501	6.2627	24.5	0.07	0.001	1.18
North Macedonia	2.9104	14.5512	24.5	4.55	0.14	3.08
Norway	2.2652	3.9131	24.8	1.20	0.011	0.90
Oman	2.5903	10.8902	24.9	2.87	0.033	1.13
Pakistan	2.6707	2.2173	24.9	0.25	0.005	2.16

## Data Availability

The data that support the present research article are included in the article.
